# Associations of exposure to volatile organic compounds with sleep health and potential mediators: analysis of NHANES data

**DOI:** 10.3389/fpubh.2024.1423771

**Published:** 2024-07-15

**Authors:** Yan Zhuang, Laifu Li, Yanqi Zhang, Fei Dai

**Affiliations:** Department of Gastroenterology, The Second Affiliated Hospital of Xi'an Jiaotong University, Xi'an, China

**Keywords:** Bayesian kernel machine regression, depression score, NHANES, sleep health, VOCs, weighted quantile sum regression

## Abstract

**Objective:**

The effect of environmental pollution on sleep has been widely studied, yet the relationship between exposure to volatile organic compounds (VOCs) and sleep health requires further exploration. We aimed to investigate the single and mixed effect of urinary VOC metabolites on sleep health and identify potential mediators.

**Methods:**

Data for this cross-sectional study was collected from the National Health and Nutrition Examination Surveys (NHANES) (2005–2006, 2011–2014). A weighted multivariate logistic regression was established to explore the associations of 16 VOCs with four sleep outcomes. Following the selection of important VOCs through the least absolute shrinkage and selection operator (LASSO) regression, principal component analyses (PCA), weight quantile sum (WQS), and Bayesian kernel machine regression (BKMR) analyses were conducted to explore the associations between exposure to single and mixed VOCs and sleep outcomes, as well as identify the most contributing components. A mediation analysis was performed to explore the potential effect of depression scores.

**Results:**

Of the 3,473 participants included in the study, a total of 618 were diagnosed with poor sleep patterns. In logistic regression analyses, 7, 10, 1, and 5 VOCs were significantly positively correlated with poor sleep patterns, abnormal sleep duration, trouble sleeping, and sleep disorders, respectively. The PCA analysis showed that PC1 was substantially linked to a higher risk of poor sleep patterns and its components. The WQS model revealed a positive association between VOC mixture of increased concentrations and poor sleep patterns [OR (95% CI): 1.285 (1.107, 1.493)], abnormal sleep duration [OR (95% CI): 1.154 (1.030, 1.295)], trouble sleeping [OR (95% CI): 1.236 (1.090, 1.403)] and sleep disorders [OR (95% CI): 1.378 (1.118, 1.705)]. The BKMR model found positive associations of the overall VOC exposure with poor sleep patterns, trouble sleeping, and sleep disorders. PCA, WQS, and BKMR models all confirmed the significant role of *N*-acetyl-*S*-(*N*-methylcarbamoyl)-l-cysteine (AMCC) in poor sleep patterns and its components. The depression score was a mediator between the positive VOC mixture index and the four sleep outcomes.

**Conclusion:**

Exposure to single and mixed VOCs negatively affected the sleep health of American population, with AMCC playing a significant role. The depression score was shown to mediate the associations of VOC mixtures with poor sleep patterns and its components.

## 1 Introduction

Sleep is a series of physiological processes regulated by neurobiology, which accounts for one-third of human life duration (1, 2). Sleep plays a crucial role in promoting health by affecting many physiological processes such as endocrine and neurological systems (3, 4). Based on an assessment of the World Health Organization, around one-third of people worldwide suffer from sleep disturbances (5). Poor sleep health is manifested by sleep disorders as well as insufficient, delayed or fragmented sleep, which is inherently sensitive to external environments such as ambient sounds, light, air quality, and environmental features around the sleep space (6). Many studies have observed an overall negative association between environmental exposures and sleep health, including heavy metals, secondhand smoke, and air pollutants, etc. (7). A cross-sectional study based on National Health and Nutrition Examination Surveys (NHANES) found that exposure to polycyclic aromatic hydrocarbons (PAHs) might be associated with poor sleep patterns (8). Previous meta-analyses have shown that self-reported exposure to secondhand smoke is positively correlated to short sleep lengths, poor sleep quality, and daytime sleepiness (9). Liu et al. (10) found that continuous exposure to air pollutants (including PM_10_, PM_2.5_, PM_1_, and NO_2_) increased the occurrence of sleep disorders while decreased sleep duration of the Chinese population. Taken together, these results suggested a possible connections between environmental exposures and sleep issues.

Volatile organic compounds (VOCs) are a combination of low-molecular-weight substances (11), including a variety of organic chemicals such as benzene and toluene, etc. (12). Both natural sources and human activities are important sources of VOCs. These compounds primarily enter the human body through inhalation or skin contact, which may affect a variety of physiological and metabolic functions of the body, such as serum lipids (13), sex hormones (14), and liver function (15), etc. One recent study found a positive correlation of co-exposure to VOCs with short sleep duration and trouble sleeping among the United States general population (16). A previous study on sewage treatment workers in the United States found that workers exposed to benzene, toluene, and other organic solvents had increased sleep requirements consistent with solvent exposure (17). A study on rats suggested that toluene exposure disrupted the sleep–wake cycle by affecting monoaminergic responses in sleep-related brain regions (18). Nowadays, cumulated evidence has indicated a close association between depression and sleep disorders, with various neurotransmitters in the central nervous system (CNS) jointly involved in emotional and sleep regulation (19). Furthermore, air pollutants are believed to affect the onset and progression of depression through inflammation and oxidative-stress-related pathways (20). Epidemiological studies have confirmed an increased risk of depression associated with VOCs (21). All these studies suggested a possible link between VOCs and sleep health, with depression potentially playing a significant role in such connections. However, current research has been mainly focused on specific occupational exposure groups limited to a few types of VOCs, with the assessment of sleep health confined to a single dimension and a lack of an exploration of its underlying mechanisms. Considering the complex interactions among VOCs and the importance of incorporating various sleep components in the analyses (22), further investigation into the combined effects of VOCs on sleep and their potential mechanisms appears essential.

Therefore, we conducted a cross-sectional study based on 3 cycles of the NHANES database using logistic regression, least absolute shrinkage and selection operator (LASSO) regression, principal component analysis (PCA), weight quantile sum (WQS), Bayesian kernel machine regression (BKMR) and mediated effects analyses to fully explore the associations between single and mixed exposures to VOCs and sleep health (including poor sleep patterns, abnormal sleep duration, trouble sleeping, and sleep disorders) among the American general population, as well as to explore the mediating effect of the depression score.

## 2 Methods

### 2.1 Study design and population

This cross-sectional study utilized data from the NHANES conducted during 2005–2006, 2011–2012, and 2013–2014. NHANES, an ongoing project led by the National Center for Health Statistics (NCHS), is a nationally-representative survey conducted in the U.S., which involves continuous data collection through interviews, physical examinations, and laboratory tests on the general population of the United States. For more detailed information, please visit the Website NHANES - National Health and Nutrition Examination Survey Homepage (cdc.gov).

In this study, a total of 30,279 participants were recruited from 3 NHANES cycles, of whom 16,308 were aged 20 years or above. After excluding 11,212 participants with missing urinary VOC data, 5,096 were collected. Of these participants, 19 with missing sleep data were excluded. To obtain more reliable results, we excluded 1,604 participants with missing covariates [ratio of family income to poverty (PIR), diabetes, hypertension, marital status, education level, body mass index (BMI), drinking status, serum cotinine]. Ultimately, 3,473 study participants were included in the analyses (Supplementary Figure S1).

### 2.2 Measurement of urinary VOCs

The quantification of urinary metabolites of VOCs was performed utilizing ultra-performance liquid chromatography-electrospray tandem mass spectrometry (UPLC-ESI/MSMS) (23). An Acquity UPLC® HSS T3 column (Part no. 186003540, 1.8 μm x 2.1 mm x 150 mm, Waters Inc.) was utilized for chromatographic separation. More detailed methods and information can be accessed on the NHANES website. In cases where analytes yielded results below the lower limit of detection (LLOD), a fill value was inserted in the analyte result field, calculated as LLOD/ $\sqrt{2}$ .

In our research, we included a total of 16 urinary VOC metabolites for analyses, each with a detection rate exceeding 70%. These metabolites were: *N*-acetyl-*S*-(2-carbamoylethyl)-l-cysteine (AAMA), 2-aminothiazoline-4-carboxylic acid (ATCA), *N*-acetyl-*S*-(*N*-methylcarbamoyl)-l-cysteine (AMCC), *N*-acetyl-*S*-(benzyl)-l-cysteine (BMA), *N*-acetyl-*S*-(n-propyl)-l-cysteine (BPMA), *N*-acetyl-*S*-(2-cyanoethyl)-l-cysteine (CYMA), *N*-acetyl-*S*-(2-carboxyethyl)-l-cysteine (CEMA), *N*-acetyl-*S*-(3,4-dihidroxybutyl)-l-cysteine (DHBMA), *N*-acetyl-*S*-(4-hydroxy-2-butenyl)-l-cysteine (MHBMA3), Mandelic acid (MA), Phenylglyoxylic acid (PGA), *N*-acetyl-*S*-(3-hydroxypropyl-1-methyl)-l-cysteine (HPMMA), 2-methylhippuric acid (2MHA), *N*-acetyl-*S*-(2-hydroxypropyl)-l-cysteine (2HPMA), 3-methylhippuric acid & 4-methylhippuric acid (3MHA + 4MHA) and *N*-acetyl-*S*-(3-hydroxypropyl)-l-cysteine (3HPMA). Details regarding the parent compounds, detection rates, LLOD, and distributions of these 16 urinary VOC metabolites can be found in Supplementary Table S1.

### 2.3 Poor sleep patterns, and its component assessment

The participants’ nighttime sleep length was determined by asking them, “How much sleep do you usually get at night on weekdays or workdays?,” and was categorized as normal (7–9 h/night) and abnormal (<7 h/night or >9 h/night). Based on answers to the question “Have you ever told a doctor or other health professionals that you have trouble sleeping?,” the presence of self-reported trouble sleeping was evaluated. To determine whether a sleep disorder was present, the question “Have you ever been told by a doctor or other health professionals that you have a sleep disorder?” was asked. When two or more of the following occur, it is considered a “poor sleep pattern”: an abnormal sleep duration (<7 h or >9 h), trouble sleeping, and sleep disorders (8, 24).

### 2.4 Measurement of the depression score

The depression score was obtained through the Patient Health Questionnaire-9 (PHQ-9), a face-to-face interview-based depression screening tool. The PHQ-9 consisted of nine questions, each of which was assigned a score of 0–3, and all item scores were ultimately summed to obtain a depression score ranging from 0 to 27 (25). The depression score reflected the frequency of participants’ depressive symptoms in the past 2 weeks, which was positively correlated with the severity of their depression symptoms. The sensitivity and specificity of diagnosing major depression with a PHQ-9 score ≥ 10 were 88% (26).

### 2.5 Covariates

Based on previous studies, potential covariates that might influence the association between VOCs and sleep health were included in this study (8, 27). Categorical covariates included gender (female and male), race (Mexican American, other Hispanic, non-Hispanic White, non-Hispanic Black, others), education level (less than grade 9, grade 9–11, high school graduate/GED or equivalent, some college or AA degree, college graduate or above), marital status (married, widowed, divorced, separated, never married, living with partner), PIR (<5, ≥5), BMI (<18.5, 18.5–24.9, 25.0–29.9, and ≥30), drinking status (no, moderate, and heavy), serum cotinine (low and high), diabetes (no, borderline, yes) and hypertension (no and yes). Continuous covariates included age. Non-drinkers were defined as individuals who had not consumed alcohol in the past year. In addition, women who drank an average of <4 drinks per day and men who drank an average of <5 drinks per day in the past year were defined as moderate drinkers, and the rest were defined as heavy drinkers. Environmental tobacco exposure was assessed using serum cotinine concentrations, categorized as low (≤0.015 ng/mL) and high (>0.015 ng/mL) using a cut-off of 0.015 ng/mL. Hypertension and diabetes mellitus were diagnosed through index measurements, medication use, and self-reports.

### 2.6 Statistical analysis

Given the complexity of the NHANES design, and following the NHANES survey reporting guidelines, we used subsample weights from 2-year VOCs divided by 3 as 6-year subsample weights for a better generalization of the results to the entire American population. We first conducted normality tests on continuous variables in the baseline characteristics analysis. Continuous variables following a normal distribution were expressed as weighted means (standard deviation, SD), with *t*-tests for between-group comparisons, and unweighted values (weighted proportions) for categorical variables, with *χ*^2^ tests for between-group comparisons. We standardized the urinary VOC concentrations for urinary creatinine, and further performed a logarithmic transformation for creatinine-corrected VOCs to conform to a normal distribution considering the right-skewed distribution of urinary VOCs. The correlation between the natural logarithm (ln)-transformed VOC concentrations under creatinine adjustment was computed using Pearson’s correlation coefficient.

We used survey-weighted multivariate logistic regression models to explore the relationship between single exposure to 16 VOCs and sleep health. Pearson correlation analyses suggested high correlations and collinearity among multiple VOCs, so we used multivariate-adjusted LASSO regression to screen out key variates associated with sleep health outcomes and construct optimal models (28). A 10-fold cross-validation was used to select the optimal lambda. Significant VOCs screened through LASSO regression were included in the subsequent PCA, WQS, BKMR, and mediation analyses.

We used PCA to transform our original correlated VOC variables into a series of uncorrelated principal components that captured important sources of variations, which could explain most variations in the original variables, realizing the downscaling of the screened important VOCs. Principal components with eigenvalues exceeding 1 were chosen (29) and integrated into the logistic regression model as continuous independent variables to investigate the associations between principal component scores and poor sleep patterns, along with their components.

To explore the mixed effect of VOCs on multiple sleep health outcomes, we fitted the screened important VOCs into WQS and BKMR models for analyses. Based on the characteristics of the WQS model, we assumed positive and negative directions, respectively, to explore the associations of the WQS index of VOCs with poor sleep patterns and its components, as well as the contribution of each VOC. The samples were pre-randomized into training and validation sets at a ratio of 4: 6, and bootstrap sampling with *N* = 1,000 was employed to generate robust estimates. In the BKMR model, we performed 20,000 iterations for all analyses using a Markov chain Monte Carlo method. First of all, we calculated the values of posterior inclusion probabilities (PIPs) for selected VOCs to identify those important for poor sleep patterns and its components using a threshold of 0.5. Secondly, the joint effect of VOCs was assessed by comparing VOC mixtures in different percentiles with the median mixture of VOCs. In addition, we fixed the remaining VOCs at the median to explore the dose–response relationship of a single VOC with poor sleep patterns and its components.

Finally, we conducted mediation analyses using the R package “mediation” to test the mediating role of depression scores in the VOC mixture index, poor sleep patterns and its components. The bootstrap method was used and simulations were repeated 5,000 times to estimate the mediation effect and confidence intervals (CIs), with all covariates corrected.

The analyses in this study were realized using R software (4.3.1) through the “survey,” “glmnet,” “factoextra,” “gWQS,” “bkmr,” and “mediation” packages. Statistical significance was defined as a two-tailed *p*-value of less than 0.05.

## 3 Results

### 3.1 Participant characteristics

Of the 3,473 participants, a total of 618 were diagnosed with poor sleep patterns. Participants’ survey-weighted baseline characteristics according to sleep patterns are shown in Table 1. Participants with poor sleep patterns were more likely to be older, widowed/divorced, who had higher levels of BMI and serum cotinine, whereas those without poor sleep patterns were more likely to be in a moderate drinking status, while those with diabetes and hypertension were more likely to have poor sleep patterns. To better illustrate the characteristics of the study population, we further compared the baseline characteristics of included and excluded subjects (Supplementary Table S2).

**Table 1:** Survey-weighted participant characteristics in NHANES (2005–2006, 2011–2014).

Characteristics	Overall	Poor sleep patterns		*p*-value^a^
		No	Yes	
*N*	3,473	2,855	618	
Gender				0.33
Male	1919 (53.13)	1,603 (53.71)	316 (50.44)	
Female	1,554 (46.87)	1,252 (46.29)	302 (49.56)	
Age	46.50 (16.36)	46.02 (16.64)	48.71 (14.82)	<0.01
Race				0.6
Mexican American	496 (7.27)	426 (7.58)	70 (5.85)	
Other Hispanic	235 (4.90)	189 (4.96)	46 (4.60)	
Non-Hispanic White	1,664 (72.27)	1,353 (71.99)	311 (73.55)	
Non-Hispanic Black	774 (9.96)	625 (9.81)	149 (10.65)	
Other Race	304 (5.60)	262 (5.66)	42 (5.35)	
Education				0.14
Less than 9th grade	306 (4.49)	256 (4.38)	50 (5.01)	
9-11th grade	458 (9.33)	368 (9.09)	90 (10.47)	
High school graduate/GED or equivalent	765 (21.84)	622 (22.05)	143 (20.88)	
Some college or AA degree	1,035 (31.24)	824 (30.40)	211 (35.11)	
College graduate or above	909 (33.10)	785 (34.09)	124 (28.53)	
Marital status				<0.01
Married	1795 (55.17)	1,510 (56.65)	285 (48.33)	
Widowed	233 (4.52)	180 (4.35)	53 (5.32)	
Divorced	385 (10.54)	276 (8.89)	109 (18.15)	
Separated	99 (2.12)	77 (2.17)	22 (1.90)	
Never married	673 (19.25)	575 (19.63)	98 (17.49)	
Living with partner	288 (8.41)	237 (8.32)	51 (8.81)	
PIR				0.73
<5	2,703 (71.52)	2,210 (71.35)	493 (72.29)	
≥5	770 (28.48)	645 (28.65)	125 (27.71)	
BMI				<0.01
<18.5	50 (1.33)	46 (1.43)	4 (0.86)	
18.5–24.9	1,001 (29.71)	870 (31.27)	131 (22.54)	
25.0–29.9	1,158 (33.48)	982 (34.48)	176 (28.88)	
≥30	1,264 (35.48)	957 (32.82)	307 (47.71)	
Drinking status				0.04
No	724 (16.19)	562 (15.36)	162 (20.04)	
Moderate	2,276 (69.08)	1903 (70.27)	373 (63.60)	
Heavy	473 (14.73)	390 (14.38)	83 (16.35)	
Serum cotinine (ng/mL)	63.46 (134.84)	57.61 (124.47)	90.36 (172.36)	<0.01
Diabetes				<0.01
No	2,637 (79.35)	2,225 (81.33)	412 (70.24)	
Borderline	271 (7.79)	223 (7.60)	48 (8.67)	
Yes	565 (12.86)	407 (11.07)	158 (21.09)	
Hypertension				<0.01
No	2060 (63.67)	1772 (65.78)	288 (53.96)	
Yes	1,413 (36.33)	1,083 (34.22)	330 (46.04)	

PIR, poverty-to-income ratio; BMI, body mass index. ^a^Student’s *t*-test for continuous variables and Chi-square test for categorical variables. Continuous variables are expressed as weighted means (standard deviation) and categorical variables are expressed as unweighted numbers (weighted percentages).

The parent compounds, detection rates, LLOD, and distributions of VOC concentrations are shown in Supplementary Table S1. The detection rates of the 16 VOCs included in this study were > 70%, among which urinary DHBMA had the highest median concentration (304.0 ng/mL), and urinary CYMA had the lowest (2.1 ng/mL). The Pearson’s coefficients of the 16 ln-transformed VOCs showed high correlations between HPMMA and MHBMA3 (*r* = 0.85), 2MHA and 3MHA + 4MHA (*r* = 0.84), CYMA and MHBMA3 (*r* = 0.81), 3HPMA and MHBMA3 (*r* = 0.81), as well as HPMMA and 3HPMA (*r* = 0.81), while other correlations were relatively weak. (Supplementary Figure S2).

### 3.2 Relationship of single VOCs with poor sleep patterns and its components

Table 2 demonstrates the correlation of single VOCs with poor sleep patterns and its components under survey-weighted logistic regression analysis after adjusting all covariates. Poor sleep patterns were positively connected with AAMA, AMCC, CEMA, DHBMA, 3HPMA, MHBMA3, and PGA. Additionally, a positive correlation was found between AAMA, AMCC, CEMA, CYMA, DHBMA, 3HPMA, MA, MHBMA3, PGA, HPMMA and abnormal sleep duration, while a negative correlation was discovered between BPMA and abnormal sleep duration. Only CEMA was positively correlated with trouble sleeping. Significant positive correlations were found between AAMA, CEMA, DHBMA, MA, MHBMA3 and sleep disorders (all P-FDR <0.05).

**Table 2:** Multivariable logistic regression analysis between single urinary VOC metabolites, poor sleep patterns, and its components.

VOCs	Poor sleep pattern		Abnormal sleep duration		Trouble sleeping		Sleep disorders	
	OR (95%CI)	FDR	OR (95%CI)	FDR	OR (95%CI)	FDR	OR (95%CI)	FDR
2MHA	1.096(0.946, 1.269)	0.305	1.094(0.988, 1.212)	0.108	1.074(0.943,1.224)	0.387	1.167(0.967,1.408)	0.137
3MHA + 4MHA	1.071(0.936, 1.225)	0.401	1.070(0.967,1.183)	0.220	1.056(0.931,1.198)	0.464	1.150(0.946,1.399)	0.187
AAMA	**1.282(1.118, 1.471)**	**0.009**	**1.151(1.031,1.284)**	**0.026**	1.147(1.011,1.301)	0.092	**1.419(1.123,1.793)**	**0.028**
AMCC	**1.333(1.102, 1.613)**	**0.020**	**1.284(1.151,1.432)**	**0.001**	1.224(1.059,1.416)	0.068	1.366(1.067, 1.749)	0.051
ATCA	1.034(0.935, 1.144)	0.565	1.024(0.949,1.105)	0.557	0.995(0.896,1.105)	0.929	0.983(0.817,1.181)	0.845
BMA	0.987(0.834, 1.169)	0.876	1.048(0.936, 1.173)	0.458	0.983(0.867, 1.114)	0.884	1.200(0.997,1.446)	0.096
BPMA	0.982(0.900, 1.071)	0.713	**0.933(0.876,0.994)**	**0.048**	0.989(0.914,1.071)	0.830	1.021(0.907,1.150)	0.820
CEMA	**1.343(1.178, 1.532)**	**0.002**	**1.166(1.057,1.286)**	**0.010**	**1.219(1.095,1.357)**	**0.016**	**1.411(1.148,1.733)**	**0.018**
CYMA	1.088(1.004, 1.179)	0.073	**1.077(1.031,1.124)**	**0.008**	1.095(1.018,1.179)	0.071	1.096(0.991,1.212)	0.105
DHBMA	**1.344(1.084, 1.665)**	**0.030**	**1.259(1.060,1.497)**	**0.023**	1.233(1.032,1.473)	0.075	**1.633(1.097,2.431)**	**0.048**
2HPMA	1.053(0.925, 1.199)	0.517	0.985(0.891,1.089)	0.759	1.053(0.955,1.161)	0.375	0.971(0.788,1.196)	0.825
3HPMA	**1.207(1.050, 1.388)**	**0.029**	**1.155(1.044, 1.278)**	**0.017**	1.140(1.032,1.258)	0.065	1.212(0.996,1.475)	0.088
MA	1.217(0.985, 1.504)	0.107	**1.171(1.033, 1.326)**	**0.025**	1.172(0.996,1.379)	0.098	**1.511(1.229,1.858)**	**0.007**
MHBMA3	**1.169(1.023, 1.335)**	**0.048**	**1.128(1.047, 1.216)**	**0.010**	1.115(1.003,1.238)	0.101	**1.226(1.036,1.451)**	**0.046**
PGA	**1.237(1.084, 1.411)**	**0.016**	**1.188(1.102,1.280)**	**0.001**	1.081(0.964,1.211)	0.275	1.348(1.064,1.707)	0.063
HPMMA	1.212(1.029,1.426)	0.053	**1.220(1.133, 1.313)**	**<0.001**	1.141(1.003,1.298)	0.090	1.201(1.007,1.433)	0.085

Models were adjusted for age, sex, race, body mass index, serum cotinine, drinking status, marital status, education level, the ratio of family income to poverty, diabetes, and hypertension. The bold number indicates the FDR < 0.05. FDR, false discovery rate; VOCs, volatile organic compounds; OR, odds ratios; CI, confidence interval.

### 3.3 Lasso regression to identify VOCs associated with poor sleep patterns and its components

Considering the high correlation among multiple VOCs, we used LASSO regression to screen out the VOCs that were more important for poor sleep patterns and its components. Based on the logarithm of *λ*, we plotted partial likelihood deviance (binomial deviance) curves and determined the optimal *λ* values for poor sleep patterns, abnormal sleep duration, trouble sleeping as well as sleep disorders to be 0.004236 [log(*λ*) = −5.464], 0.005043 [log(*λ*) = −5.290], 0.003715 [log(*λ*) = −5.595] and 0.004153 [log(*λ*) = −5.484] respectively (Supplementary Figure S3). The contraction coefficient curves were further plotted to select VOCs that were more correlated with the dependent variables. For poor sleep patterns, a total of 10 VOCs (AAMA, AMCC, BMA, BPMA, CEMA, CYMA, 2HPMA, MHBMA3, PGA, HPMMA) were included in the analyses; 3MHA + 4MHA, AMCC, ATCA, BPMA, CEMA, CYMA, 2HPMA, PGA, and HPMMA were more associated with abnormal sleep duration; for trouble sleeping, AMCC, BMA, BPMA, CEMA, CYMA, DHBMA, and 2HPMA were included; while AMCC, ATCA, DHBMA, MA, and PGA were considered more relevant to sleep disorders (Supplementary Figure S4).

### 3.4 Principal component analysis (PCA) on VOC mixtures

We identified 2, 2, 2, 1 PCs for poor sleep patterns, abnormal sleep duration, trouble sleeping, and sleep disorders using eigenvalues through PCA analyses, which, respectively, explained 59.59, 57.37, 59.34, and 49.49% of the total variance in the VOC exposure (Supplementary Table S3). Supplementary Figure S5 depicts the loadings of each selected VOC on the principal components. For poor sleep patterns, the first PC exhibited similar moderate variable loadings for AAMA, AMCC, CEMA, CYMA, MHBMA3, and HPMMA in the same direction. PC2 showed a high positive load fraction of BPMA, while negative loadings for CYMA, AMCC, MHBMA3, AAMA, and HPMMA. Notably, AMCC had high positive loadings in all the four sleep outcomes of PC1.

In the principal component analysis adjusting all covariates, PC1 was significantly positively associated with poor sleep patterns [OR (95% CI): 1.114 (1.044, 1.188), *p* = 0.002], while PC2 showed no significant association [OR (95% CI): 0.929 (0.800, 1.078), *p* = 0.315]. In the principal component analysis on abnormal sleep duration, both PC1 and PC2 were significantly and positively associated with the risk of abnormal sleep duration [OR (95% CI): 1.095 (1.053, 1.139), *p* < 0.001 and 1.127 (1.040, 1.221), *p* = 0.006 respectively]. Principal component analysis on trouble sleeping showed that PC1 was positively associated with the risk of trouble sleeping [OR (95% CI): 1.169 (1.052, 1.298), *p* = 0.006], whereas PC2 was not significantly associated with it [OR (95% CI): 1.030 (0.862, 1.231), *p* = 0.734]. In terms of sleep disorders, PC1 had a significant positive association with sleep disorders [OR (95% CI): 1.246 (1.113, 1.394), *p* < 0.001] (Table 3).

**Table 3:** Association between VOCs, poor sleep patterns, and its components: principal component analysis results.

	OR	95% CI	*p*-value
Poor sleep patterns			
PC1	1.114	(1.044, 1.188)	0.002
PC2	0.929	(0.800, 1.078)	0.315
Abnormal sleep duration			
PC1	1.095	(1.053, 1.139)	<0.001
PC2	1.127	(1.040, 1.221)	0.006
Trouble sleeping			
PC1	1.169	(1.052, 1.298)	0.006
PC2	1.030	(0.862, 1.231)	0.734
Sleep disorders			
PC1	1.246	(1.113, 1.394)	<0.001

Models were adjusted for age, sex, race, body mass index, serum cotinine, drinking status, marital status, education level, the ratio of family income to poverty, diabetes, and hypertension. CI, confidence interval; OR, odds ratios; PC, principal component; VOCs, volatile organic compounds.

### 3.5 WQS analysis of single and mixed VOCs with poor sleep patterns, and its components

**Figure 1:**
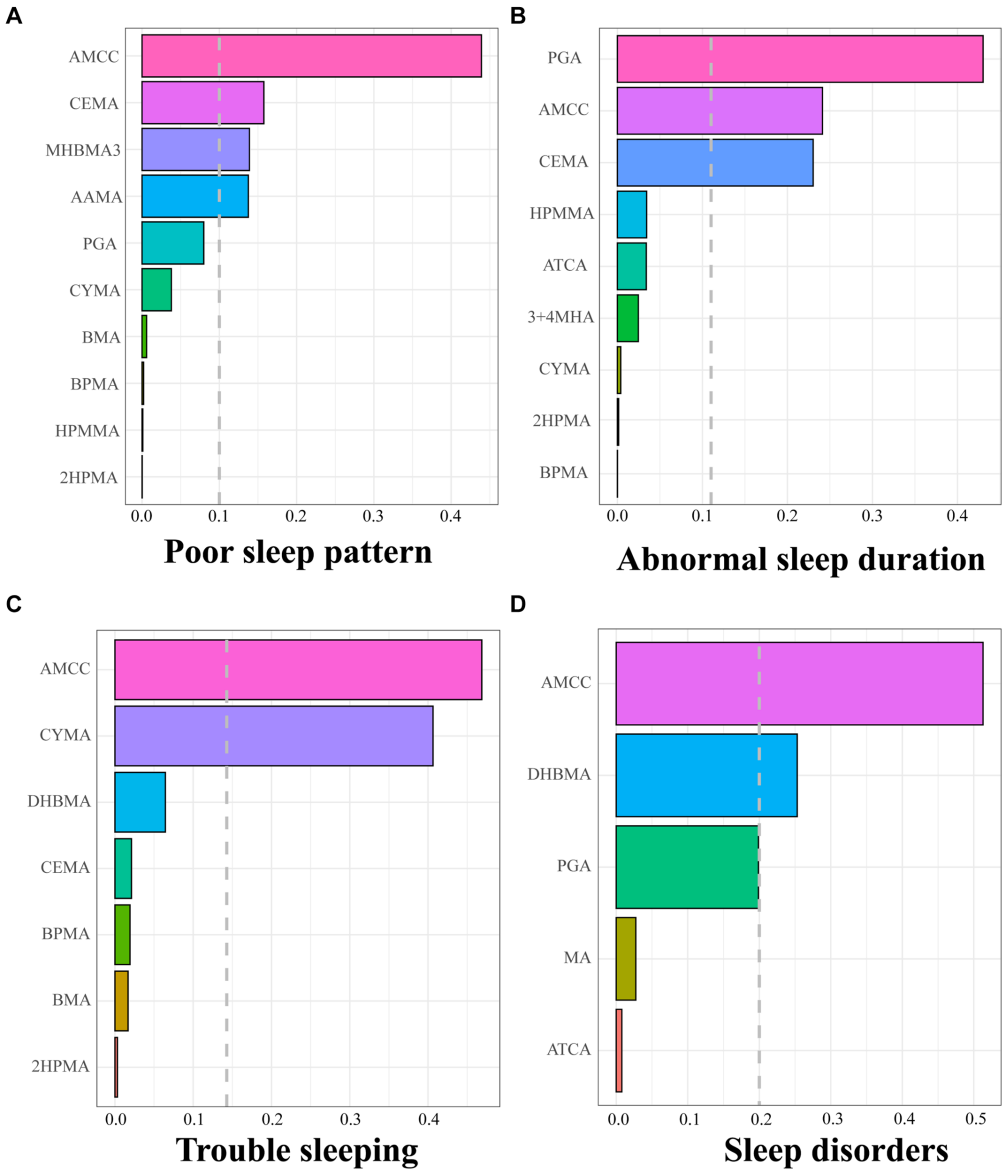
Positive weights of WQS index of screened urinary VOCs in poor sleep pattern **(A)**, abnormal sleep duration **(B)**, trouble sleeping **(C)**, and sleep disorder **(D)**. The dashed grey lines represent the cutoff to discriminate which element has a significant weight. Models were adjusted for age, sex, race, body mass index, serum cotinine, drinking status, marital status, education level, the ratio of family income to poverty, diabetes, and hypertension.

We constructed WQS regression models to explore the relationship between important VOCs screened based on LASSO and sleep health. As shown in Supplementary Table S4, the WQS index of urinary VOC mixture showed a significant positive correlation with poor sleep patterns [OR (95% CI): 1.285(1.107, 1.493)], abnormal sleep duration [OR (95% CI): 1.154(1.030, 1.295)], trouble sleeping [OR (95% CI): 1.236(1.090, 1.403)] and sleep disorders [OR (95% CI): 1.378(1.118, 1.705)]. Figure 1 shows that AMCC, CEMA, MHBMA3, and AAMA are major contributors to poor sleep patterns, PGA, AMCC, and CEMA are significant for abnormal sleep duration, AMCC and CYMA are key for trouble sleeping, while AMCC and DHBMA are primary contributors to sleep disorders. In addition, we also investigated the negative associations of the mixture of VOCs with sleep health while did not find a substantial negative association between the combined VOC WQS index and poor sleep patterns, or its components (Supplementary Table S5 and Supplementary Figure S6).

### 3.6 BKMR analysis of single and mixed VOCs with poor sleep patterns, and its components

**Figure 2:**
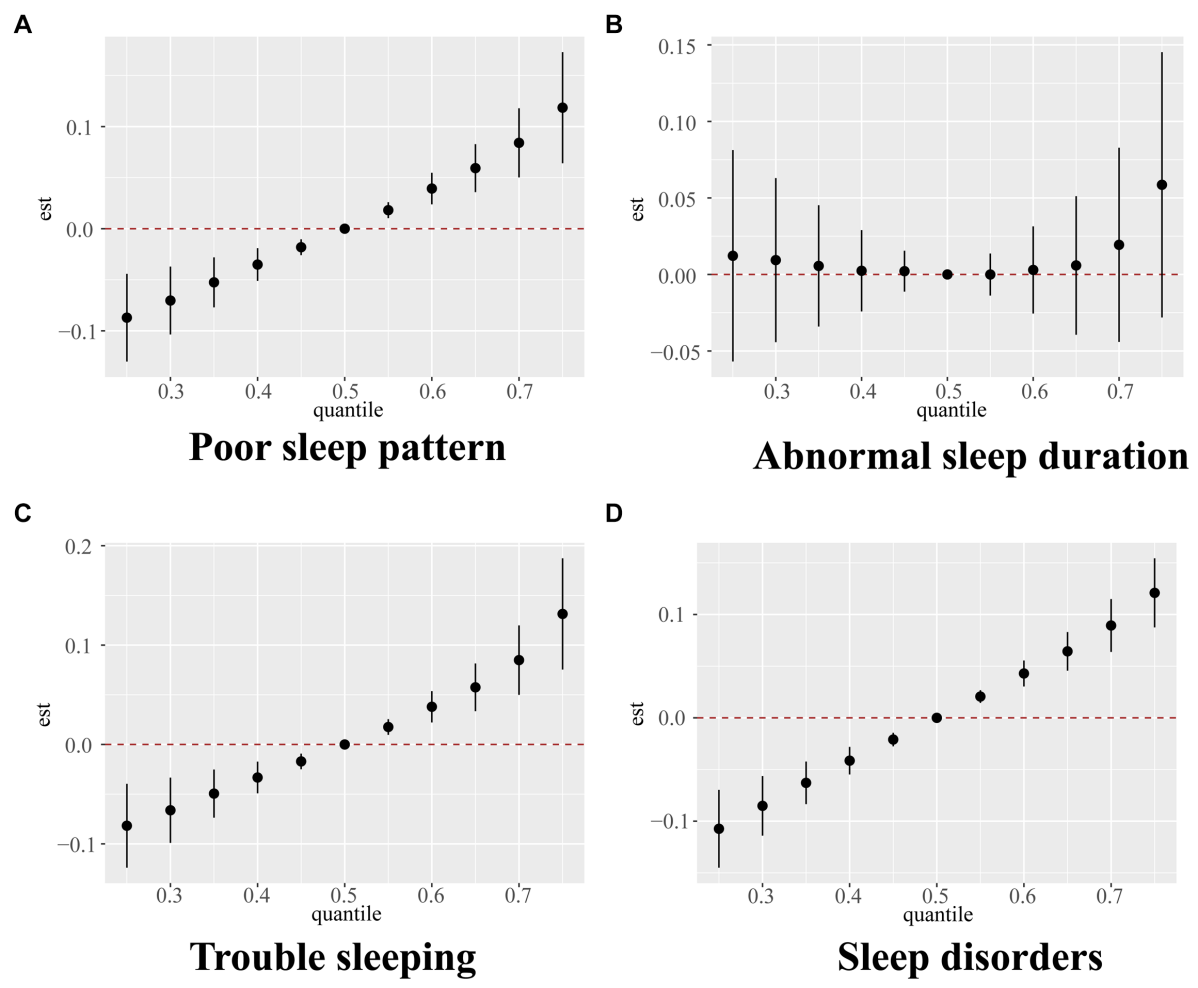
Overall relationship between the mixture of VOCs and poor sleep pattern **(A)**, abnormal sleep duration **(B)**, trouble sleeping **(C)**, and sleep disorder **(D)** estimated by Bayesian kernel machine regression (BKMR) model. Models were adjusted for age, sex, race, body mass index, serum cotinine, drinking status, marital status, education level, the ratio of family income to poverty, diabetes, and hypertension.

The screened key VOCs were incorporated into the BKMR model to further validate the mixture effect of VOCs on sleep health. Supplementary Table S6 shows the PIP values of selected VOCs, among which AMCC had the highest PIP value in terms of poor sleep patterns, trouble sleeping, and sleep disorders. AMCC, BPMA, CYMA, 2HPMA, and PGA made great contributions to abnormal sleep duration. Figure 2 shows the overall effect of VOC mixtures and their estimated changes in the risk of poor sleep patterns as well as its components, compared to those when all VOCs are fixed at the median. We found that the overall effect of urinary VOCs was significantly and positively associated with poor sleep patterns, trouble sleeping, and sleep disorders when all VOCs were above the 55th percentile. Further, exposure-response relationships between single VOCs and sleep health indicators were analyzed when fixing the remaining VOCs at the 50th percentile level. We found that AMCC was significantly nonlinearly and positively correlated with poor sleep patterns, trouble sleeping, and sleep disorders. BPMA and 2HPMA were non-linearly and negatively correlated, while CYMA and PGA were nonlinearly and positively correlated with abnormal sleep duration. In addition, we also observed a nonlinear association between AMCC and abnormal sleep duration (Supplementary Figure S7).

### 3.7 Mediation analysis of the relationship between VOCs mixture index and poor sleep patterns, and its components

**Figure 3:**
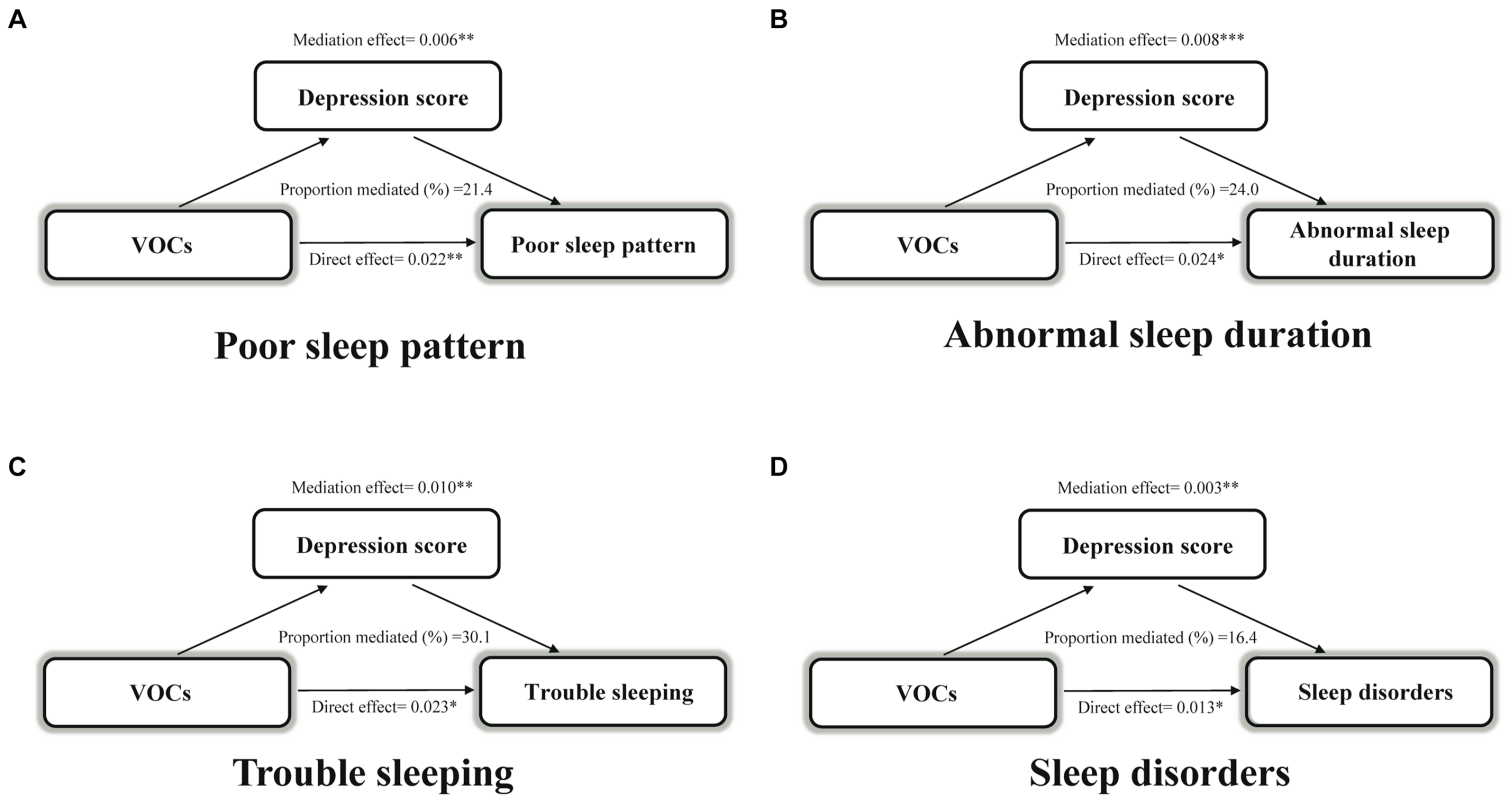
Estimated proportion of the association between VOCs mixture index and poor sleep pattern **(A)**, abnormal sleep duration **(B)**, trouble sleeping **(C)**, and sleep disorder **(D)** mediated by depression score. Models were adjusted for age, sex, race, body mass index, serum cotinine, drinking status, marital status, education level, the ratio of family income to poverty, diabetes, and hypertension. ^*^p < 0.05; ^**^p < 0.01; ^***^p < 0.001. VOCs, volatile organic compounds.

Next, we explored whether VOCs indirectly affected sleep health through depression scores. Depression scores mediated 21.4, 24.0, 30.1, and 16.4% of the associations of VOC mixture index with poor sleep patterns, abnormal sleep duration, trouble sleeping, and sleep disorders, respectively, (all *p* < 0.05) (Figure 3).

## 4 Discussion

For the first time, our study provided the systematic and comprehensive confirmation of the relationship between VOCs and various sleep outcomes. According to single and mixed models, AMCC was consistently positively correlated with poor sleep patterns, abnormal sleep duration, trouble sleeping, and sleep disorders. In mixed analyses, PCA, WQS, and BKMR models supported that co-exposure to VOCs was significantly and positively associated with poor sleep patterns, trouble sleeping, and sleep disorders. In addition, depression scores mediated the associations of co-exposure to VOCs with poor sleep patterns and its components.

Limited studies have investigated the effect of exposure to VOCs on sleep health. A survey on the general population of the United States revealed that with increasing co-exposure to VOCs, the risks of short sleep duration and trouble sleeping significantly elevated (16). Thetkathuek et al. (30) found that workers exposed to xylene and toluene were more likely to experience drowsiness compared to those not exposed to solvents, with the lack of personal protective equipment being a major factor affecting sleep disorders. Several studies on rats suggested that central monoaminergic mechanisms were associated with toluene-induced partial insomnia and sleep–wake cycle disruption (18, 31). A cross-sectional study showed a higher prevalence of sleep disturbances among tunnel workers previously exposed to acrylamide and *N*-methylolacrylamide (32). In addition, considering that tobacco smoke is a major source of VOC exposure, the relationship between secondhand smoke exposure and sleep health is partly suggestive of the impact of VOCs on sleep. A large number of cross-sectional and cohort studies have shown that exposure to secondhand smoke is significantly associated with poor sleep health such as poor sleep quality, sleep maintenance disorders, and short sleep duration (33, 34), which supports our speculation. However, current relevant studies are limited to certain single VOC exposures, which lack a comprehensive assessment of sleep outcomes, making it difficult to generalize the results.

In real life, people are commonly exposed to a variety of mixed VOCs, making a comprehensive assessment on the impact of VOCs on sleep of significant public health importance. In this study, to address the collinearity and correlation issues among multiple VOCs, we conducted PCA, WQS, and BKMR analyses based on LASSO regression to better capture the combined toxic effects of VOC exposure on sleep health outcomes. These models also supplemented and corroborated the findings of logistic regression on individual VOCs and health outcomes, aiming to identify exposure components that contribute more significantly to the outcomes. In our study, all analytical models pointed to an elevated incidence of poor sleep patterns, as well as its components with increasing concentrations of AMCC. AMCC was considered a key triggering factor for poor sleep outcomes. As a major component of AMCC, dimethylformamide (DMF) is a widely-used drug solvent; however, the mechanism through which DMF induces poor sleep outcomes is unclear. Notably, in terms of the BKMR exposure-response function, we found that when the metabolites of other VOCs were fixed at the median level, AMCC showed a nonlinear relationship with poor sleep patterns and its components. Specifically, as the concentration of AMCC increased, the prevalence of poor sleep patterns and its components initially decreased and then increased, with this non-linear association being more pronounced in abnormal sleep duration. A plausible explanation for this is that DMF has a wide range of CNS depressant effect, which enhances a pentobarbitone-induced increase in sleep duration (35, 36). A moderate increase in sleep duration promotes normal metabolism and homeostasis, while a continued accumulation of AMCC leading to excessive sleep duration results in poor sleep outcomes and harms the body (37).

Currently, it is unclear how exposure to VOCs influences sleep disturbances. Chronic exposure may affect sleep outcomes through CNS regulation and physiologic changes in the respiratory system. First of all, air pollutants may lead to an altered and dysregulated expression of neurochemicals through the CNS. Specifically, it has been demonstrated that air pollution lowers the serotonin level in the brain (38). Serotonin is one of the most important brain chemicals that regulate the sleep–wake cycle. A decrease in the serotonin level can result in drowsiness and lead to sleep disturbances (39). Several *in vitro* studies have shown that exposure to high levels of VOCs promotes oxidative stress in human lung epithelial cells, and the reactive oxygen species (ROS)-induced activation of pro-inflammatory genes and transcription factors triggers the production of inflammatory mediators (40), which leads to respiratory-related sleep disturbances and reduces sleep quality (7). Furthermore, inflammatory signals reach the CNS through active mechanisms and cellular pathways involved in direct neural innervation, the effect of humoral mediators, and blood–brain barrier transport, thereby influencing alterations in sleep patterns (41). Given the complex additive and synergistic effects among various VOCs, more experimental and epidemiological studies will be needed in the future to elucidate the underlying mechanisms.

Previous studies have shown that urinary VOC concentrations are significantly positively correlated with depressive symptoms (21). A cross-sectional study based on NHANES 2007–2014 found a positive dose–response connection between clinically-relevant depression and sleep patterns (42). About 90% of depressed patients complain of sleep quality problems such as insomnia (43), while sleep quality problems or depression do not occur at the same time, whose emergence is dependent on many other factors (44). Based on the above studies, we hypothesized that depression could be a potential mechanism between VOC exposure and sleep health. Further mediation analyses also validated this speculation: depression scores mediated 21.4, 24.0, 30.1, and 16.4% of the associations of VOCs with poor sleep patterns, abnormal sleep duration, trouble sleeping, and sleep disorders, respectively. At the mechanistic level, VOCs promote the generation of oxidative-stress-mediated inflammatory mediators in the body, leading to a state of systemic chronic inflammatory stress and an increasing depression risk (21). Similar changes in neurotransmitter receptor systems and neuroendocrine responses between depression and sleep disturbances may play a significant role in their relationship. One hypothesis suggested that depression was caused by an imbalance between cholinergic and monoaminergic neurotransmitter production, which was closely related to the regulation of rapid eye movement (REM) sleep (19). Other studies have suggested that dysfunctions of the orexin system may be related to the pathophysiology of mood regulation and the sleep–wake cycle (45). In addition, depression inhibits melatonin secretion, interferes with circadian rhythms, and disrupts sleep (46). In conclusion, our findings provided evidence that improving depressive symptoms might reduce the negative effects of VOC exposure on sleep health. Based on the above research results, we advocate for the government to strengthen the management of VOC concentrations by enacting stricter regulations. Public awareness of VOCs should be raised in the future, with attention to personal protection. Furthermore, the role of mental and emotional well-being in the impact of environmental pollution on physical health should be emphasized to achieve better health management.

There are several strengths of our study. Firstly, based on the NHANES database, we were able to explore our study on a large sample population. Secondly, we utilized LASSO regression to select the most relevant VOCs associated with four sleep outcomes, addressing multicollinearity issues arising from highly-correlated variables. Additionally, we employed various mixed-effect models to complement each other, confirming the mixed negative effects of VOCs on poor sleep outcomes. Of note, we identified AMCC as a potential compound closely related to poor sleep patterns and its components, providing insights for further research into the mechanisms underlying adverse sleep outcomes.

Our study also has certain limitations. Firstly, since self-reporting was the basis for sleep health outcomes in this study, recall biases or inaccurate reporting could potentially skew the findings. It will be necessary in the future to classify sleep issues through objective tests such as actigraphy for a more refined assessment. Secondly, urinary VOC metabolites were assessed based on a single measurement without exposure timing information in NHANES. This measurement method can only represent current VOC levels and may lead to measurement errors. Thirdly, our study had inherent limitations as a cross-sectional study, which prevented us from establishing a causal relationship between VOC exposure and sleep outcomes. More cohort studies and randomized controlled trials are needed in the future to better reveal this association.

## 5 Conclusion

Single and combined VOC exposure increased the risk of poor sleep patterns, abnormal sleep duration, trouble sleeping, and sleep disorders, with AMCC being a significant contributor. Depression scores mediated the associations between VOC mixtures and sleep outcomes. Our study emphasizes the potential of controlling VOC exposure for improving sleep health, and advocates for the future formulation of health policies regarding VOC regulation to normalize VOC concentration management. In the future, additional prospective research will be required to validate and expand upon our findings.

## Data availability statement

The raw data supporting the conclusions of this article will be made available by the authors, without undue reservation.

## Ethics statement

The studies involving humans were approved by CDC’s NCHS Ethics Review Board. The studies were conducted in accordance with the local legislation and institutional requirements. The participants provided their written informed consent to participate in this study.

## Author contributions

YZhu: Conceptualization, Data curation, Formal analysis, Methodology, Writing – original draft. LL: Writing – review & editing. YZha: Writing – review & editing. FD: Supervision, Writing – review & editing.

## Glossary

2HPMA: *N*-acetyl-*S*-(2-hydroxypropyl)-l-cysteine
2MHA: 2-methylhippuric acid
3HPMA: *N*-acetyl-*S*-(3-hydroxypropyl)-l-cysteine
3MHA + 4MHA: 3-methylhippuric acid & 4-methylhippuric acid
AAMA: *N*-acetyl-*S*-(2-carbamoylethyl)-l-cysteine
AMCC: *N*-acetyl-*S*-(*N*-methylcarbamoyl)-l-cysteine
ATCA: 2-aminothiazoline-4-carboxylic acid
BKMR: Bayesian kernel machine regression
BMA: *N*-acetyl-*S*-(benzyl)-l-cysteine
BMI: Body mass index
BPMA: *N*-acetyl-*S*-(n-propyl)-l-cysteine
CEMA: *N*-acetyl-*S*-(2-carboxyethyl)-l-cysteine
CI: Confidence interval
CNS: Central nervous system
CYMA: *N*-acetyl-*S*-(2-cyanoethyl)-l-cysteine
DHBMA: *N*-acetyl-*S*-(3,4-dihidroxybutyl)-l-cysteine
DMF: Dimethylformamide
FDR: False discovery rate
HPMMA: *N*-acetyl-*S*-(3-hydroxypropyl-1-methyl)-l-cysteine
LASSO: Least absolute shrinkage and selection operator
Ln: Natural logarithm
LLOD: Lower limit of detection
MA: Mandelic acid
MHBMA3: *N*-acetyl-*S*-(4-hydroxy-2-butenyl)-l-cysteine
NHANES: National Health and Nutrition Examination Survey
OR: Odds ratios
PAHs: Polycyclic aromatic hydrocarbons
PCA: Principal component analysis
PGA: Phenylglyoxylic acid
PHQ-9: Patient Health Questionnaire-9
PIPs: Posterior inclusion probabilities
PIR: Ratio of family income to poverty
ROS: Reactive oxygen species
SD: Standard deviation
VOCs: Volatile organic compounds
WQS: Weighted quantile sum
