# Supplementary material for: Associations of exposure to volatile organic compounds with sleep health and potential mediators: analysis of NHANES data
Source: Front Public Health. 2024 Jul 15;12:1423771. doi: 10.3389/fpubh.2024.1423771 (PMC11284068; doi:10.3389/fpubh.2024.1423771)
Supplement: Supplementary file 1 [file Data_Sheet_1.docx]

Supplementary Material

**Tables:**

**Table S1 Distribution of VOC metabolites (N = 3473), NHANES, USA, 2005-2006, 2011–2014.**

| **VOC metabolites(ng/mL)** | **Abbreviation** | **Parent Compound** | **Detection rate** | **LLOD** | **Geometric mean** | **Median（25th-75th）** |
| --- | --- | --- | --- | --- | --- | --- |
| 2-methylhippuric acid | 2MHA | Xylene | 94.56% | 5 | 35.51 | 34.9(15.7, 82.2) |
| 3-methylhippuric acid & 4-methylhippuric acid | 3MHA+4MHA | Xylene | 99.54% | 8 | 252.86 | 248.0 (108.0, 599.0) |
| N-acetyl-S-(2-carbamoylethyl)-L-cysteine | AAMA | Acrylamide | 99.37% | 2.2 | 54.04 | 54.3 (28.6, 106.0) |
| N-acetyl-S-(N-methylcarbamoyl)-L-cysteine | AMCC | N, N-Dimethylformamide | 99.22% | 6.26 | 159.19 | 161.0(81.3, 336.0) |
| 2-aminothiazoline-4-carboxylic acid | ATCA | Cyanide | 83.24% | 15 | 83.96 | 101.0(35.6, 217.0) |
| N-acetyl-S-(benzyl)-L-cysteine | BMA | Toluene | 99.19% | 0.5 | 7.27 | 7.1(3.7, 13.6) |
| N-acetyl-S-(n-propyl)-L-cysteine | BPMA | 1-Bromopropane | 73.28% | 1.2 | 4.21 | 3.8(0.9, 10.8) |
| N-acetyl-S-(2-carboxyethyl)-L-cysteine | CEMA | Acrolein | 98.88% | 6.96 | 99.52 | 104.0(52.0, 195.0) |
| N-acetyl-S-(2-cyanoethyl)-L-cysteine | CYMA | Acrylonitrile | 91.19% | 0.5 | 4.80 | 2.1(1.0, 26.9) |
| N-acetyl-S-(3,4-dihidroxybutyl)-L-cysteine | DHBMA | 1,3-Butadiene | 99.71% | 5.25 | 275.99 | 304.0(169.0, 492.0) |
| N-acetyl-S- (2-hydroxypropyl)-L-cysteine | 2HPMA | Propylene oxide | 94.99% | 5.3 | 35.42 | 34.8(17.8, 67.4) |
| N-acetyl-S- (3-hydroxypropyl)-L-cysteine | 3HPMA | Acrolein | 99.42% | 13 | 267.22 | 265.0(129.0, 538.0) |
| Mandelic acid | MA | Styrene | 98.82% | 12 | 140.59 | 146.0(81.0, 252.0) |
| N-acetyl-S- (4-hydroxy-2-butenyl)-L-cysteine | MHBMA3 | 1,3-Butadiene | 97.75% | 0.6 | 6.83 | 6.2(3.1, 14.5) |
| Phenylglyoxylic acid | PGA | Ethylbenzene, styrene | 91.94% | 12 | 143.30 | 184.0(79.7, 334.0) |
| N-acetyl-S-(3-hydroxypropyl-1-methyl)-L-cysteine | HPMMA | Crotonaldehyde | 99.88% | 1.7 | 277.45 | 255.0(137.0, 529.0) |

**Notes:** VOC, volatile organic compound; LLOD, lower limit of detection.

**Table S2 Comparison of the characteristics of the subsample included and excluded in this study from the NHANES (2005-2006, 2011-2014).**

| **Characteristics** | **Subsample included in this study** | **Subsample excluded in this study** |  |
| --- | --- | --- | --- |
|  |  |  |  |
| **N** | 3473 | 26806 |  |
| **Gender** |  |  |  |
| Male | 1919 (55.25) | 13020 (48.57) |  |
| Female | 1554 (44.75) | 13786 (51.43) |  |
| **Age** | 48.22 (17.51) | 27.94 (24.21) |  |
| **Race** |  |  |  |
| Mexican American | 496 (14.28) | 5436 (20.28) |  |
| Other Hispanic | 235 (6.77) | 2150 (8.02) |  |
| Non-Hispanic White | 1664 (47.91) | 8911 (33.24) |  |
| Non-Hispanic Black | 774 (22.29) | 6886 (25.69) |  |
| Other Race | 304 (8.75) | 3423 (12.77) |  |
| **Education** |  |  |  |
| Less than 9th grade | 306 (8.81) | 1327 (10.34) |  |
| 9-11th grade | 458 (13.19) | 1881 (14.66) |  |
| High school graduate/GED or equivalent | 765 (22.03) | 2888 (22.50) |  |
| Some college or AA degree | 1035 (29.80) | 3809 (29.68) |  |
| College graduate or above | 909 (26.17) | 2909 (22.66) |  |
| Refused | 0 (0.00) | 10 (0.08) |  |
| Don't know | 0 (0.00) | 11 (0.09) |  |
| **Marital status** |  |  |  |
| Married | 1795 (51.68) | 6564 (45.10) |  |
| Widowed | 233 (6.71) | 1133 (7.78) |  |
| Divorced | 385 (11.09) | 1316 (9.04) |  |
| Separated | 99 (2.85) | 446 (3.06) |  |
| Never married | 673 (19.38) | 4039 (27.75) |  |
| Living with partner | 288 (8.29) | 1037 (7.13) |  |
| Refused | 0 (0.00) | 16 (0.11) |  |
| Don't know | 0 (0.00) | 3 (0.02) |  |
| **PIR** |  |  |  |
| <5 | 2703 (77.83) | 21203 (86.03) |  |
| ≥5 | 770 (22.17) | 3444 (13.97) |  |
| **BMI** |  |  |  |
| <18.5 | 50 (1.44) | 5482 (23.70) |  |
| 18.5-24.9 | 1001 (28.82) | 7339 (31.73) |  |
| 25.0-29.9 | 1158 (33.34) | 5059 (21.87) |  |
| ≥30 | 1264 (36.40) | 5252 (22.70) |  |
| **Drinking status** |  |  |  |
| No | 724 (20.85) | 1937 (21.72) |  |
| Moderate | 2276 (65.53) | 5695 (63.87) |  |
| Heavy | 473 (13.62) | 1284 (14.40) |  |
| **Serum cotinine (ng/mL)** | 64.42 (138.63) | 34.82 (100.26) |  |
| **Diabetes** |  |  |  |
| No | 2637 (75.93) | 21730 (87.00) |  |
| Borderline | 271 (7.80) | 991 (3.97) |  |
| Yes | 565 (16.27) | 2257 (9.04) |  |
| **Hypertension** |  |  |  |
| No | 2060 (59.31) | 14561 (72.97) |  |
| Yes | 1413 (40.69) | 5395 (27.03) |  |

**Notes: Continuous variables are expressed as means (standard deviation) and categorical variables are expressed as numbers (percentages). All data are presented in an unweighted form, and the analyses do not consider missing data. PIR, poverty-to-income ratio; BMI, body mass index.**

**Table S3 Main characteristics of each principal component**

|  | **eigenvalue** | **cumulative variance  percent (%)** |
| --- | --- | --- |
| **Poor sleep patterns** |  |  |
| PC1 | 4.73 | 47.34 |
| PC2 | 1.22 | 59.59 |
| **Abnormal sleep duration** |  |  |
| PC1 | 3.87 | 43.05 |
| PC2 | 1.29 | 57.37 |
| **Trouble sleeping** |  |  |
| PC1 | 2.97 | 42.41 |
| PC2 | 1.18 | 59.34 |
| **Sleep disorders** |  |  |
| PC1 | 2.47 | 49.49 |

**Notes:** **Models were adjusted for** age, sex, race, body mass index, serum cotinine, drinking status, marital status, education level, the ratio of family income to poverty, diabetes, and hypertension. PC, **principal component.**

**Table S4 Association between WQS indices, poor sleep patterns, and its components in the positive direction**

| **Outcomes** | **OR** | **95% CI** | **P-value** |
| --- | --- | --- | --- |
| Poor sleep pattern | 1.285 | (1.107, 1.493) | 0.001 |
| Abnormal sleep duration | 1.154 | (1.030, 1.295) | 0.014 |
| Trouble sleeping | 1.236 | (1.090, 1.403) | <0.001 |
| Sleep disorders | 1.378 | (1.118, 1.705) | 0.003 |

**Notes:** **Models were adjusted for** age, sex, race, body mass index, serum cotinine, drinking status, marital status, education level, the ratio of family income to poverty, diabetes, and hypertension. OR, odds ratios; CI, confidence interval.

**Table S5 Association between WQS index, poor sleep patterns, and its components in the negative direction**

| **Outcomes** | **OR** | **95% CI** | **P-value** |
| --- | --- | --- | --- |
| Poor sleep pattern | 0.966 | (0.833, 1.120) | 0.644 |
| Abnormal sleep duration | 0.958 | (0.871,1.054) | 0.381 |
| Trouble sleeping | 1.013 | (0.887, 1.157) | 0.844 |
| Sleep disorders | 1.005 | (0.847, 1.194) | 0.950 |

**Notes:** **Models were adjusted for** age, sex, race, body mass index, serum cotinine, drinking status, marital status, education level, the ratio of family income to poverty, diabetes, and hypertension. OR, odds ratios; CI, confidence interval.

**Table S6 PIP values in BKMR**

|  | **Poor sleep patterns** | **Abnormal sleep duration** | **Trouble sleeping** | **Sleep disorders** |
| --- | --- | --- | --- | --- |
| **2MHA** | - | - | - | - |
| **3MHA+ 4MHA** | - | 0.3014 | - | - |
| **AAMA** | 0.0002 | - | - | - |
| **AMCC** | 1 | 0.7369 | 0.9862 | 1 |
| **ATCA** | - | 0.3204 | - | 0.0085 |
| **BMA** | 0.0028 | - | 0.0177 | - |
| **BPMA** | 0.0232 | 0.6049 | 0.0221 | - |
| **CEMA** | 0.0111 | 0.4096 | 0.0039 | - |
| **CYMA** | 0.0147 | 0.5447 | 0.0797 | - |
| **DHBMA** | - | - | 0.0003 | 0.0014 |
| **2HPMA** | 0 | 0.5136 | 0.0057 | - |
| **3HPMA** | - | - | - | - |
| **MA** | - | - | - | 0.0005 |
| **MHBMA3** | 0.0004 | - | - | - |
| **PGA** | 0.0028 | 0.7167 | - | 0.0076 |
| **HPMMA** | 0.0004 | 0.3474 | - | - |

**Notes:** PIPs, posterior inclusion probabilities; -, not involved in the analysis.

**Figures:**

**Fig. S1: Flowchart of participants included in the final analysis (N=3473), NHANES, USA, 2005-2006, 2011-2014.**

**
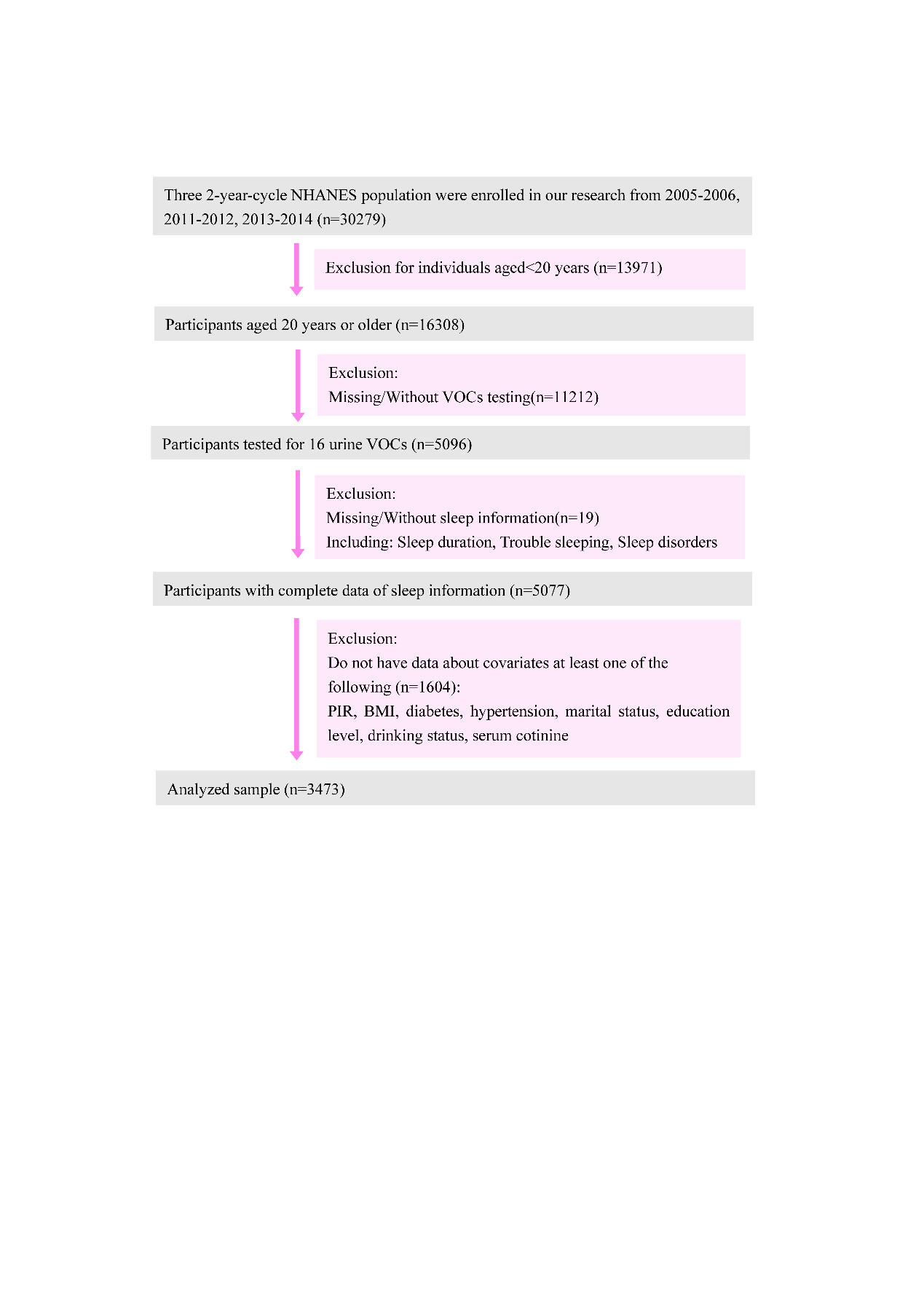
**

**Notes:** VOCs, volatile organic compounds; PIR, poverty income ratio; BMI, body mass index.

**Fig. S2: Pearson correlation coefficient between urinary VOCs.**


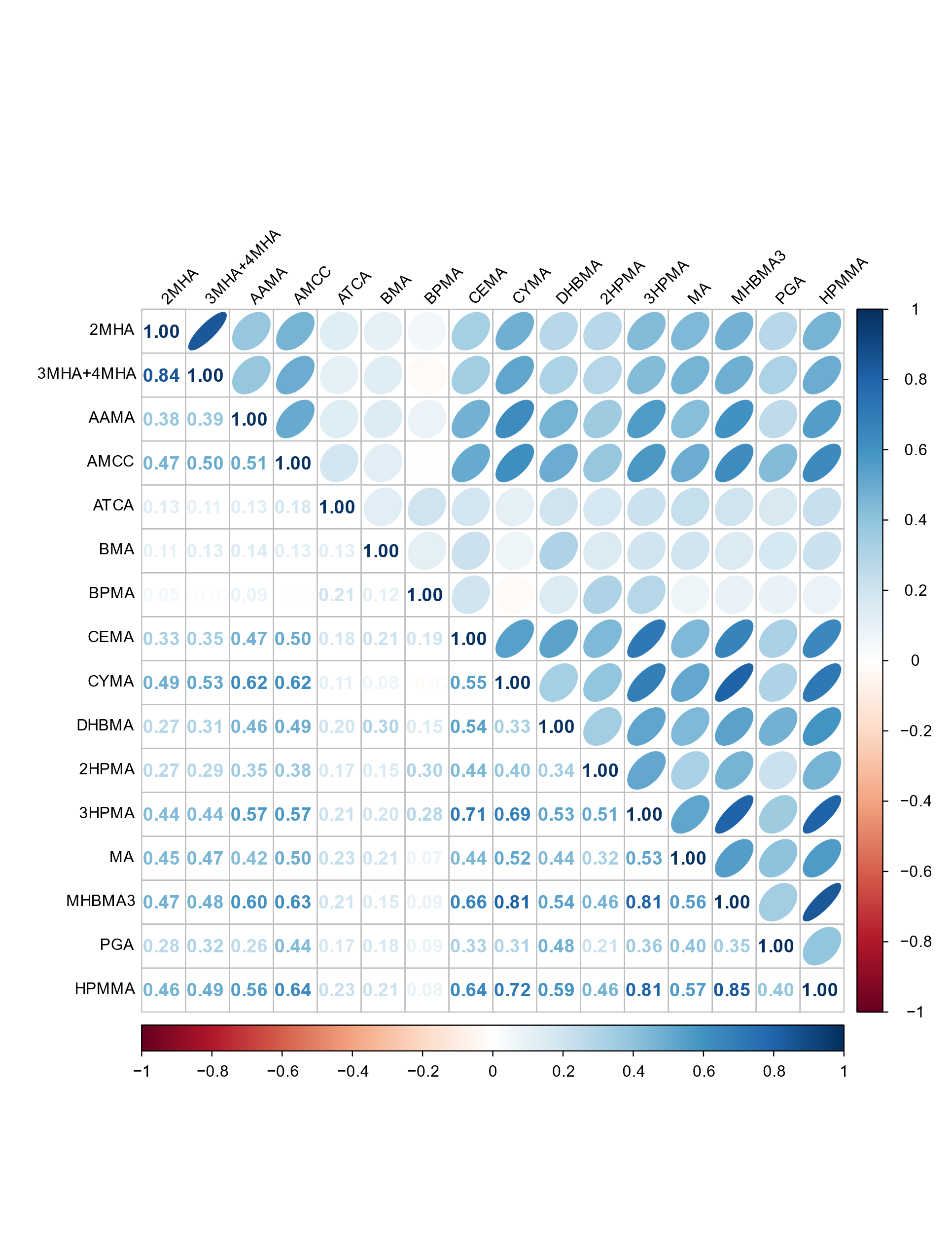


**Fig. S3: 10-fold cross-validation curves between log-transformed LASSO penalty parameter (λ) and variables.**


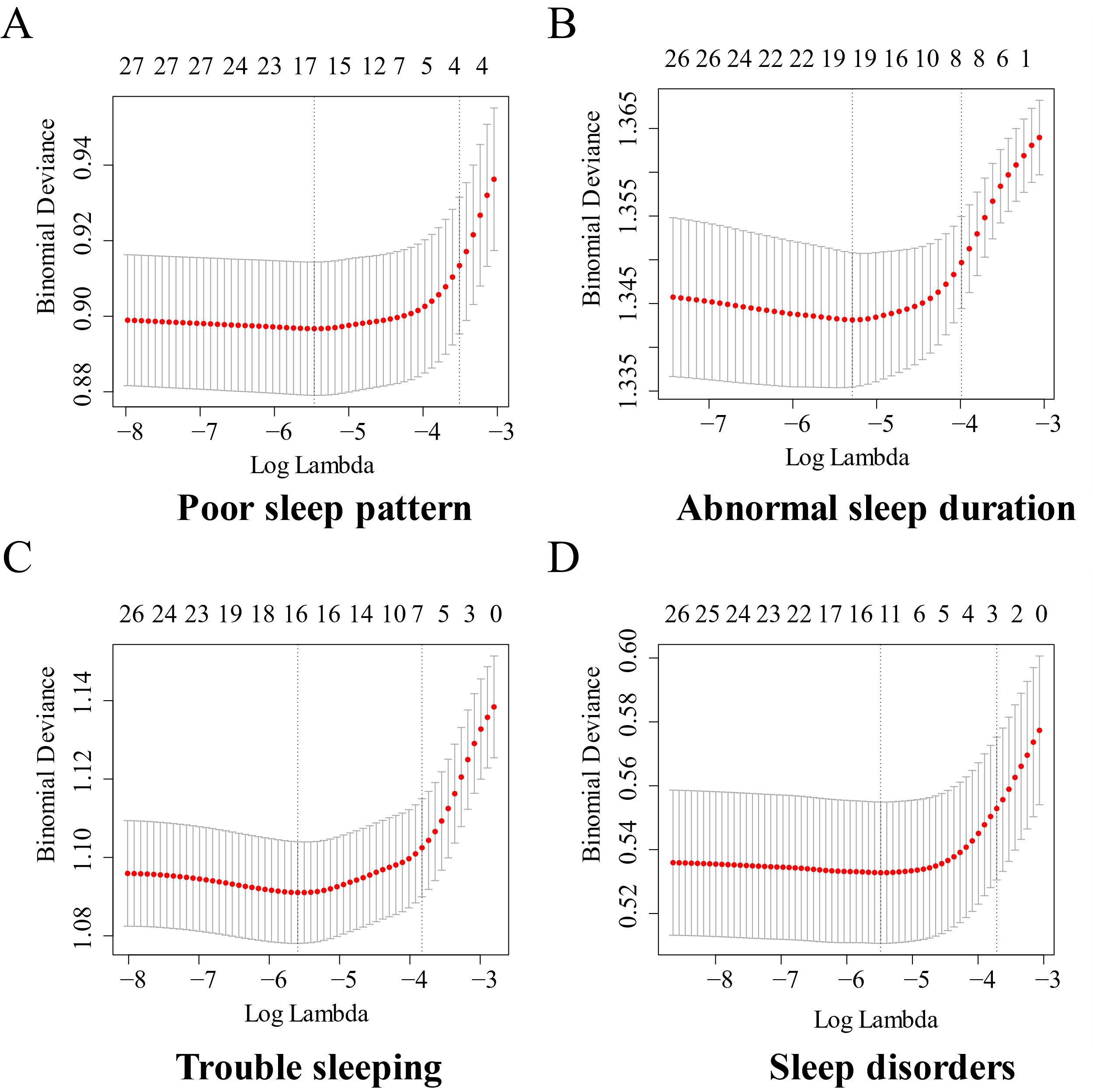


**Notes:** The independent variables are ln-transformed 16 VOCs corrected with creatinine. Covariates include age, sex, race, body mass index, serum cotinine, drinking status, marital status, education level, the ratio of family income to poverty, diabetes, and hypertension. Poor sleep pattern (**A**), abnormal sleep duration (**B**), trouble sleeping (**C**), and sleep disorder (**D**) are studied as dependent variables, respectively. The two dashed lines represent the simplest model for the optimal λ (left) and the simplest model for λ that is within one standard error of the optimal value (right).

**Fig S4: Shrinkage coefficient curves between the log-transformed LASSO penalty parameter (λ) and the variates.**


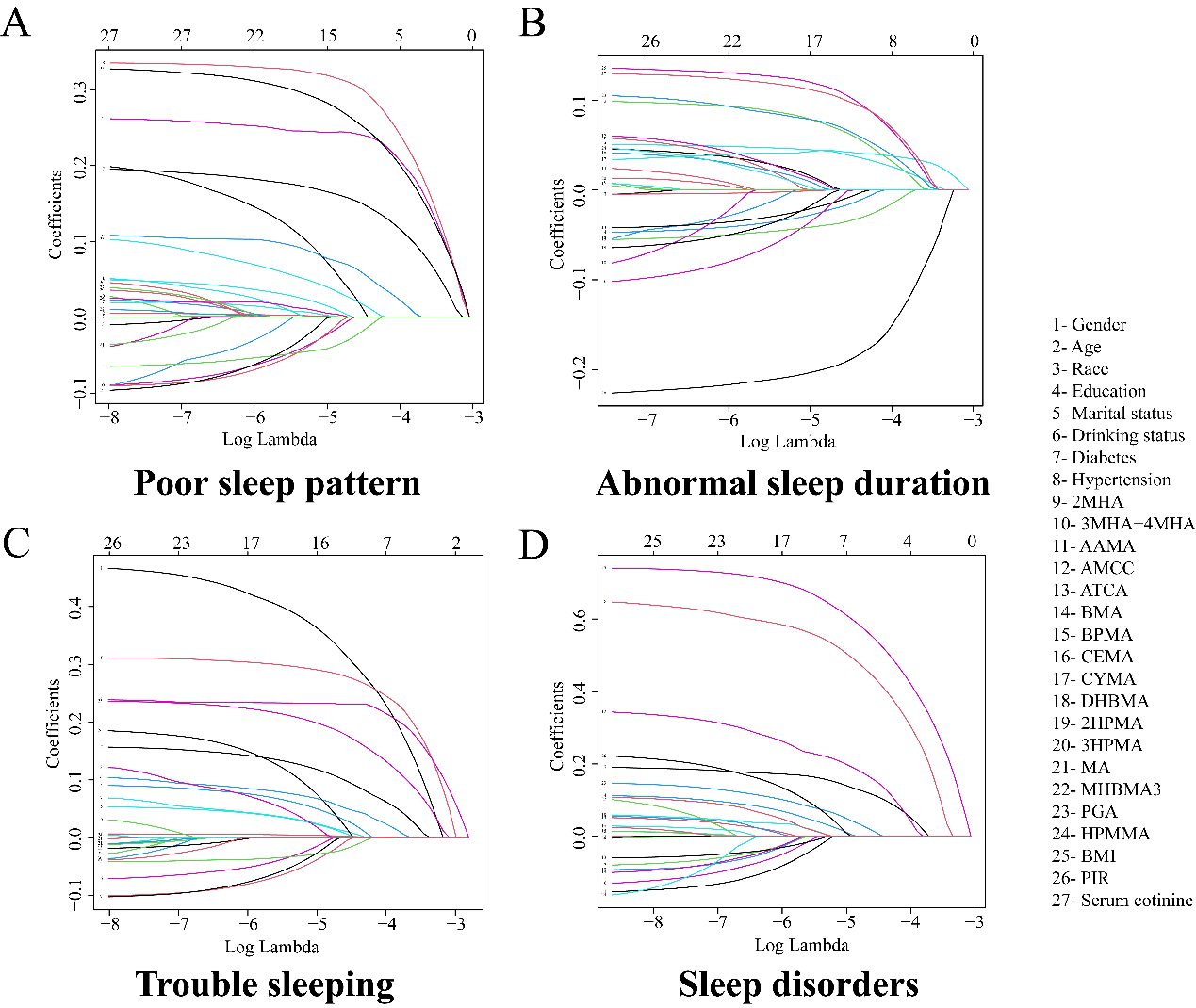


**Notes:** The independent variables are ln-transformed 16 VOCs corrected with creatinine. Covariates include age, sex, race, body mass index, serum cotinine, drinking status, marital status, education level, the ratio of family income to poverty, diabetes, and hypertension. Poor sleep pattern (**A**), abnormal sleep duration (**B**), trouble sleeping (**C**), and sleep disorder (**D**) are studied as dependent variables, respectively. PIR, poverty-to-income ratio; BMI, body mass index.

**Fig. S5: Specific loadings of each selected VOC related to the initial principal component in principal component analysis.**


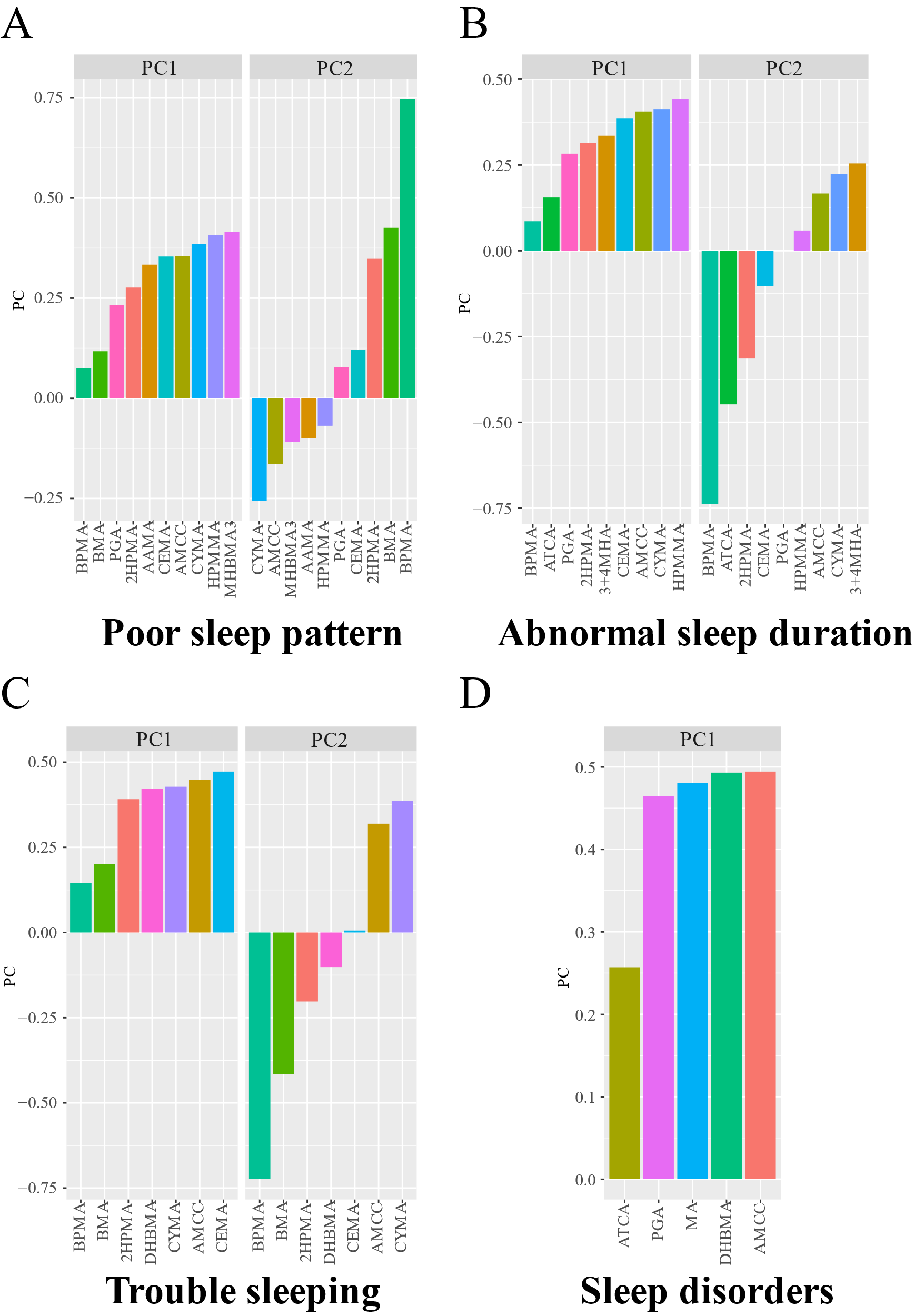


**Fig. S6: Negative weights of WQS index of screened urinary VOCs in poor sleep pattern (A), abnormal sleep duration (B), trouble sleeping (C), and sleep disorder (D).**


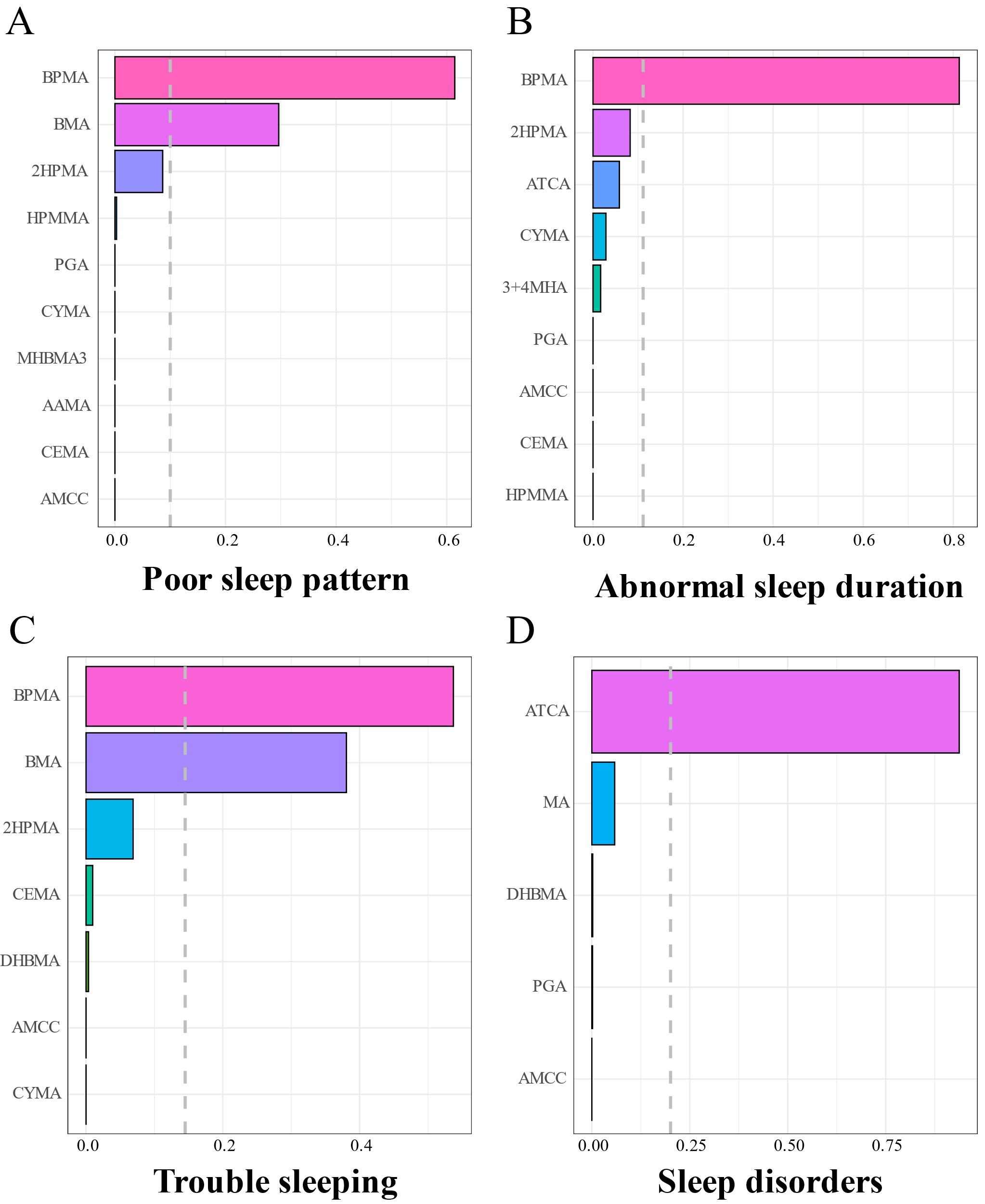


**Notes:** The dashed grey lines represent the cutoff to discriminate which element has a significant weight. Models were adjusted for age, sex, race, body mass index, serum cotinine, drinking status, marital status, education level, the ratio of family income to poverty, diabetes, and hypertension.

**Fig S7: Univariate exposure-response functions between screened volatile organic compounds and poor sleep pattern (A), abnormal sleep duration (B), trouble sleeping (C), and sleep disorder (D) estimated by the BKMR model.**


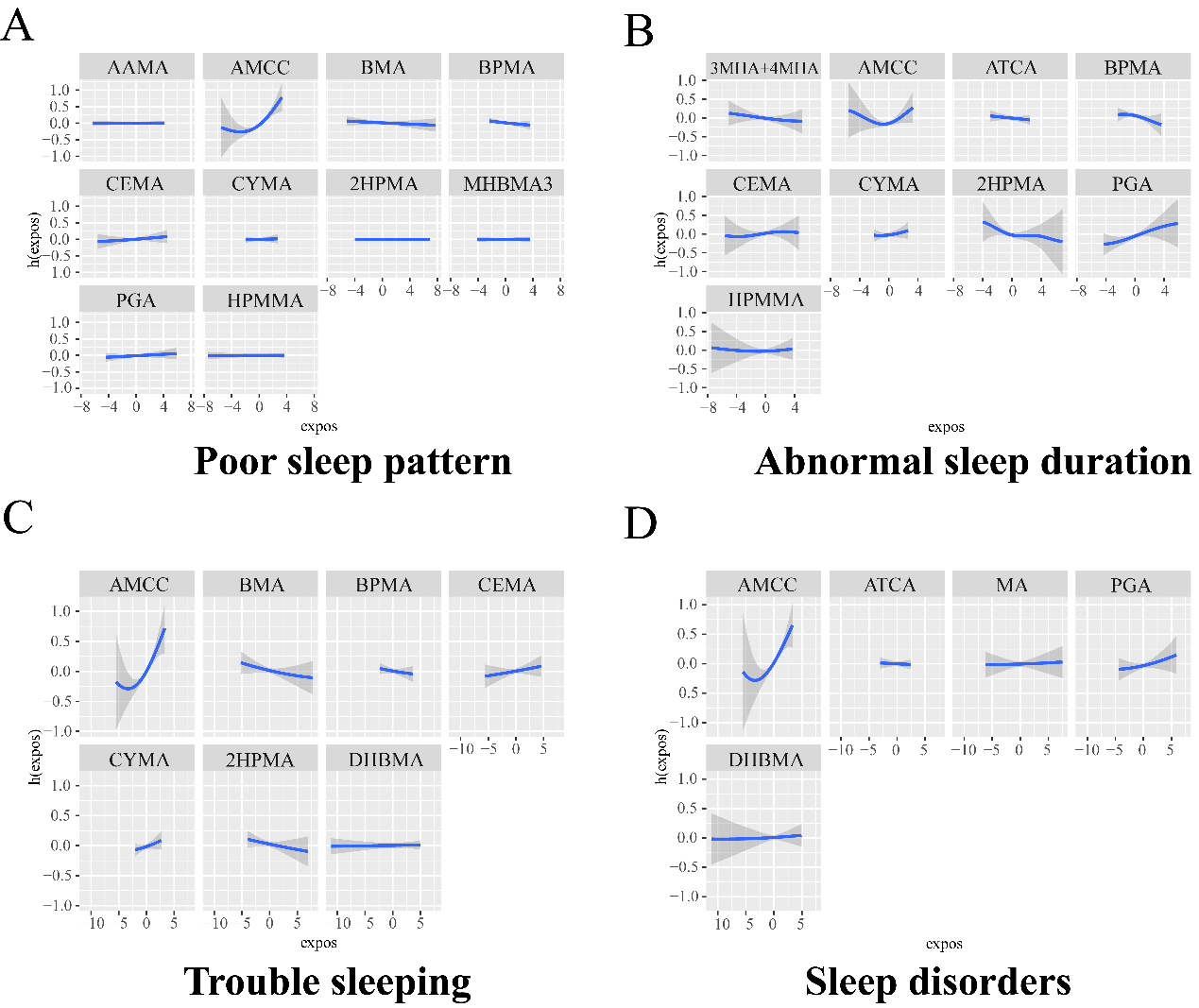


**Notes:** Models were adjusted for age, sex, race, body mass index, serum cotinine, drinking status, marital status, education level, the ratio of family income to poverty, diabetes, and hypertension.
